# Phytochemicals Mediate Autophagy Against Osteoarthritis by Maintaining Cartilage Homeostasis

**DOI:** 10.3389/fphar.2021.795058

**Published:** 2021-12-20

**Authors:** Zheng Tian, Xinan Zhang, Mingli Sun

**Affiliations:** School of Kinesiology, Shenyang Sport University, Shenyang, China

**Keywords:** autophagy, phytochemicals, Osteoarthritis, chondrocytes, inflammation

## Abstract

Osteoarthritis (OA) is a common degenerative joint disease and is a leading cause of disability and reduced quality of life worldwide. There are currently no clinical treatments that can stop or slow down OA. Drugs have pain-relieving effects, but they do not slow down the course of OA and their long-term use can lead to serious side effects. Therefore, safe and clinically appropriate long-term treatments for OA are urgently needed. Autophagy is an intracellular protective mechanism, and targeting autophagy-related pathways has been found to prevent and treat various diseases. Attenuation of the autophagic pathway has now been found to disrupt cartilage homeostasis and plays an important role in the development of OA. Therefore, modulation of autophagic signaling pathways mediating cartilage homeostasis has been considered as a potential therapeutic option for OA. Phytochemicals are active ingredients from plants that have recently been found to reduce inflammatory factor levels in cartilage as well as attenuate chondrocyte apoptosis by modulating autophagy-related signaling pathways, which are not only widely available but also have the potential to alleviate the symptoms of OA. We reviewed preclinical studies and clinical studies of phytochemicals mediating autophagy to regulate cartilage homeostasis for the treatment of OA. The results suggest that phytochemicals derived from plant extracts can target relevant autophagic pathways as complementary and alternative agents for the treatment of OA if subjected to rigorous clinical trials and pharmacological tests.

## 1 Introduction

Osteoarthritis (OA) is a common degenerative joint disease occurring in the elderly population and is the most common cause of pain and disability worldwide. Its pathology is characterized by progressive cartilage degeneration, increased subchondral bone remodeling and bone redundancy formation (Kraus et al., 2015), which poses a great burden to the patient’s family and society (Nelson, 2018; Jones et al., 2019). The main pathological features of OA include articular cartilage erosion, synovitis and subchondral bone degeneration (Hunter and Bierma-Zeinstra, 2019), but the exact mechanisms are not known. Available studies suggest that multiple pathological factors such as overloading, trauma, imbalance of inflammatory systems, and impairment of anti-inflammatory pathways are involved in the development and progression of OA, leading to cellular senescence, reduced cell density, abnormal secretory activity, extracellular matrix (ECM) degradation, and impaired articular cartilage development (Goldring and Otero, 2011; Sofat et al., 2011; Doria et al., 2013; Robinson et al., 2016; Li et al., 2017b; Khan et al., 2018; Wang and He, 2018).

Conservative treatment is currently the main treatment for OA and is mainly limited to pain control. Various drugs, such as Nonsteroidal anti-inflammatory drugs (NSAIDs), cyclooxygenase-2 (COX-2) inhibitors, glucosamine, steroids, and hyaluronic acid (Philp et al., 2017) have been used clinically to slow the progression of OA, but they are limited to pain control and do not reverse the effects of OA, and all have significant side effects with long-term use (Bert and Bert, 2014; Cutolo et al., 2015; Hunter and Bierma-Zeinstra, 2019). Joint replacement is the mainstay of treatment for advanced knee OA, and although it is effective, it is expensive and has a limited lifespan (Zhang et al., 2016; Nelson, 2018; Kloppenburg and Berenbaum, 2020). Therefore, there is an urgent need to explore new means of OA treatment.

Autophagy is a self-protective mechanism (Benderdour et al., 2015; Sohn et al., 2017) that relies on autophagosomes and lysosomes to efficiently maintain internal environment homeostasis by removing protein aggregates or unwanted cellular components (Zhi et al., 2018). Under hypoxia, nutritional deficiency, endoplasmic reticulum stress (ERS), or other pathological conditions, autophagy is activated to degrade dysfunctional intracellular components, thereby improving cell survival and function (Li et al., 2019b). Currently, targeting autophagy-related pathways has been shown to be a new approach to treating diseases such as liver diseases and tumors, and has received widespread attention from researchers (Togano et al., 2021; Zhou et al., 2021a). During the development of OA, decreased chondrocyte autophagy leads to impaired cellular function, resulting in joint aging and dysfunction (Netea-Maier et al., 2016), indicating that autophagy plays an important role in the development of OA and is expected to be an important target for OA treatment.

Recently, relevant studies have found that phytochemicals play an important role in disease prevention and treatment by mediating the autophagic pathway (Zhang et al., 2015; Ranjan et al., 2019; Mao et al., 2020). In recent decades, due to the effectiveness of phytochemicals in disease prevention and treatment, botanical drugs and natural products are widely used as complementary and alternative medicines for the treatment of OA to function (Cameron and Chrubasik, 2014) and have received increasing attention in the last decade. Naturally sourced phytochemicals are both widely available and inexpensive, and provide a valuable source of lead compounds or adjuvant components for the development of new drugs against OA. In recent years, many studies (Chin and Pang, 2017; Jiang et al., 2020; Luk et al., 2020) have shown that phytochemicals derived from plants or botanical drugs may play an important role in the prevention or treatment of OA by activating autophagy through different mechanisms. However, there is no comprehensive review article reporting preclinical and clinical studies of phytochemicals improving OA by mediating autophagy-related pathways. Given the important role of autophagy in the progression of OA, we carefully reviewed phytochemicals from different plant sources that may help regulate autophagy-related signaling pathways and can be used to treat OA through improved cartilage. We searched the scientific literature for the last decade systematically using the authoritative internet databases of PubMed, Web of Science, and Embase by combining the keywords “autophagy”, “osteoarthritis”, and “plant”. The primary search criterion was the application of phytochemicals of different plant origin for the treatment of OA through autophagy. These phytochemicals can be classified as polyphenols, flavonoids, terpenoids, coumarins, saponins, and small molecule compounds. Table 1 gives an overview of the resource profile and mechanisms of phytochemicals that mediate the autophagic pathway against OA, with species and family names according to the relevant literature based on www.theplantlist.org, http://www.plantsoftheworldonline.org/, http://mpns.kew.org/mpns-portal/ (Rivera et al., 2014).

**TABLE 1:** Phytochemicals improve cartilage homeostasis via mediated autophagy against OA *in vitro/in vivo*.

	Phytochemical	Plant species, family	Model	Dosage range	Active concentration	Signal pathways/Mechanisms	References
Polyphenols	Curcumin	*Curcuma longa* L., Zingiberaceae Martinov	*In vitro*, IL-1β induced rats chondrocytes	5 μM, 10 μM, 15 μM, and 20 μM	10 μM	MAPK/ERK1/2 signal pathway	Li et al. (2017a)
			*In vitro*, IL-1β induced rats chondrocytes	1.25–20 μM	10 μM	—	Chen et al. (2021)
			*In vivo*, DMM surgery mice	50 mg/kg *via* oral administration, once daily for 8 weeks	50 mg/kg	AKT/mTOR pathway	Zhang et al. (2018a)
			*In vitro,* IL-1β induced rats chondrocytes	10 μM	10 μM		
			*In vivo*, HFD rats	200 μg/kg body weight and 400 μg/kg body weight *via* joint injection	200 μg/kg body weight	E2F1/PITX1 pathway and AKT/mTOR pathway	Yao et al. (2021)
	Hydroxytyrosol	*Olea europaea* L., Oleaceae Hoffmanns. and Link	*In vitro*, TNF-αmice chondrocytes	0, 12.5, 25, 50, 100, 200 and 400 μM, last for 24 h	50 μM	SIRT6 pathway	Zhi et al. (2018)
			*In vitro*, H_2_O_2_ induced OA human chondrocytes	100 μM for 30 min	100 μM	SIRT1 pathway	Cetrullo et al. (2016)
	Resveratrol	*Vitis vinifera* L., Vitaceae Juss	*In vitro*, DMM surgery mice chondrocytes	125 mg *via* intra-articular injection for 8 w	125 mg	HIF-1*α*-dependent AMPK and mTOR signaling	Qin et al. (2017)
			*In vivo*, DMM surgery mice				
	Butein	*Butea monosperma (Lam.)* Kuntze, Fabaceae Lindl	*In vitro*, IL-1β induced TKA surgery human chondrocytes	0.6 μg/ml-10 μg/ml (or 2.25–36 μM) for 24 h	10 μg/ml (36 μM)	AMPK/TSC2/ULK1/mTOR pathway	Ansari et al. (2018a)
	Mangiferin	*Mangifera indica* L., Anacardiaceae R.Br	*In vivo*, DMM surgery mice	10 mg/kg once a day for 8 weeks	10 mg/kg	AMPK signaling pathway	Li et al. (2019b)
			*In vitro,* TBHP-induced chondrocytes from mice	0, 5, 10, 50, 100, 200 μM	100 μM		
	Delphinidin	*Aristotelia chilensis* (Molina) Stuntz, Elaeocarpaceae Juss	*In vitro*, H_2_O_2_ induced C28/I2 human chondrocytes	10–75 μM	40 μM	Nrf2 and NF-κB were activated	Lee et al. (2020)
	Punicalagin	*Punica granatum* L., Lythraceae J.St.-Hil	*In vitro*, TBHP induced mice chondrocytes	0–50 μg/ml	50 μg/ml	Autophagic flux in chondrocytes after TBHP treatment recovered	Kong et al. (2020)
			*In vivo,* DMM surgery mice	20 mg/kg via oral administration each day for 8 weeks	20 mg/kg		
			*In vitro,* rats chondrocytes	0, 25, 50 and 100 μM	50 μM	Foxo1/Prg4/HIF3*α* axis	Liu et al. (2021)
			*In vivo,* Rats with cut anterior cruciate ligament, medial collateral ligament and medial meniscus	10 mg/kg *via* oral administration for 12 weeks	10 mg/kg		
	(-)Epigallocatechin 3-Gallate	*Camellia sinensis* (L.) Kuntze, Theaceae Mirb. ex Ker Gawl	*In vivo,* ACLT surgery rats	10 μM EGCG by intra-articular injection once every 3 days for 5 weeks	10 μM	mTOR expression was reduced and LC3, Beclin-1 and p62 expression were increased	Huang et al. (2020)
	Chlorogenic acid	*Bauhinia macrantha* Oliv*.,* Fabaceae Lindl	*In vitro,* Human chondrocyte C28/I2 cells	0, 50, 100, 200, 250, 400 μM	250 μM	Antioxidant response proteins Nrf2 and NF-κB were increased	Zada et al. (2021)
Flavonoids	Icariin	*Epimedium sagittatum* (Sieb. and Zucc.) Maxim., Berberidaceae Juss	*In vitro*, TNF-α induced rats chondrocytes	0, 3, 5, 7, 10, and 20 μM	10 μM	p65 nuclear translocation and IκBα protein degradation were inhibited	Mi et al. (2018)
			*In vivo,* ACTL surgery rats	20, 40, or 80 mg/kg/day by intraperitoneal injection for 4 consecutive weeks	20 mg/kg	PI3K/AKT/mTOR pathway	Tang et al. (2021)
			*In vitro,* chondrocytes from ACTL surgery rats	1–100 μM	80 μM		
	Baicalin	*Scutellaria baicalensis* Georgi*,* Lamiaceae Martinov	*In vitro*, IL-1β-induced chondrocytes from TKA surgery OA patients	20 μM	20 μM	miR-766–3p level was upregulated and AIFM1 expression was decreased	Li et al. (2020)
	Glabridin	*Glycyrrhiza glabra* L*.,* Fabaceae Lindl	*In vitro,* human OA chondrocytes	0.01–10 μM	1 μM	mTOR pathway	Dai et al. (2021b)
			*In vivo,* ACLT surgery rats	1, 5, and 10 mg/kg for 4 or 8 weeks	1 mg/kg		
	Rhoifolin	*Rhus succedanea* L*.*, Anacardiaceae R.Br	*In vitro,* IL-1β induced rats chondrocytes	0, 5, 10, and 20 μM	20 μM	P38/JNK pathway and PI3K/AKT/mTOR pathway	Yan et al. (2021)
			*In vivo,* ACLT surgery rats	20 μM intra-articular injection weekly for 8 weeks	20 μM		
	Eupatilin	*Artemisia absinthium* L., Asteraceae Bercht. and J.Presl	*In vitro,* IL-1β induced rats chondrocytes	0, 25, 50, 100 μM	25 μM	Senstrin2-dependent autophagy	Lou et al. (2019)
	Sinensetin	*Citrus* L., Phyllanthaceae Martinov	*In vitro,* TBHP induced mice chondrocytes	0, 10, 20, 30, 40, and 50 μM	10 μM	AMPK/mTOR signaling pathway	Zhou et al. (2021a)
			*In vivo,* DMM surgery mice	50 mg/kg by gavage for 8 w	50 mg/kg		
Terpenoids	Morroniside	*Corni Fructus,* Cornaceae Bercht. and J.Presl	*In vitro*, TKA surgery human chondrocytes	0, 1, 20, 200 μM	20 μM	PI3K/AKT/mTOR signal pathway	Xiao et al. (2020)
	Lycopene	*Solanum lycopersicum* L., Solanaceae Juss	*In vitro,* H_2_O_2_ induced SD rats chondrocytes	0.001–10 μM	0.1 μM	MAPK and PI3K/Akt/NF-κB axis	Wu et al. (2021)
	Celastrol	*Tripterygium wilfordii,* Celastraceae R.Br	*In vitro,* IL-1β induced SD rats chondrocytes	0–1.6 μM	0.2 μM	The expression of LC3-II and Beclin-1 increased	Feng et al. (2020)
			*In vivo,* ACLT surgery rats	0.5 mg/kg, 1 mg/kg by intraperitoneal injection for 12 w	0.5 mg/kg		
Coumarins	Isoimperatorin	*Angelica dahurica*, Apiaceae Lindl	*In vivo*, DMM surgery mice	500 mg/kg *via* oral admin- istration for 4 w	500 mg/kg	mTORC1 pathway	Ouyang et al. (2017)
			*In vitro,* DMM surgery mice chondrocytes	1–100 mM	1 μM		
	Isopsoralen	*Cullen corylifolium* (L.) Medik., Fabaceae Lindl	*In vitro,* IL-1β induced rats chondrocytes	5, 10, 20, and 40 μg/ml	20 μg/ml	LC3-II and LAMP-1 expression was significantly increased, but p62/SQSTM1 expression was significantly decreased	Chen et al. (2020b)
Saponin	Astragaloside IV	*Astragalus mongholicus* Bunge, Fabaceae Lindl	*In vitro*, IL-1β induced TKA surgery human chondrocytes	50 μg/ml	50 μg/ml	Protein expression of LC3-II/I was increased and that of P62/SQSTM1 was decreased	Liu et al. (2017)
	Huzhangoside D	*Clematis graveolens* Lindl., Ranunculaceae Juss	*In vivo,* ACLT surgery rats	17, 34, 68 mg/kg *via* intraperitoneal injection daily for 4 w	17 mg/kg	AKT and mTOR signaling pathway	Zhang et al. (2021)
Small molecules compounds	*β*-ecdysterone	*Achyranthes bidentata*, Amaranthaceae Juss	*In vivo*, MIA intraperitoneal injection induced OA rats	0.6 mg/kg, 0.8 mg/kg, and 1 mg/kg *via* subcutaneous injection twice a week for 4 weeks	0.6 mg/kg	PI3K/AKT/MTOR signal pathway	Tang et al. (2020)
			*In vitro,* DMM surgery mice chondrocytes	10, 20, 40 μM	10 μM		
	Dihydroartemisinin	*Artemisia annua* L., Asteraceae Bercht. and J.Presl	*In vitro,* TNF-α induced rats chondrocytes	0–10 μM	1 μM	LC3-II and ATG5 levels were increased and the expression of MMP-3 and -9, ADAMTS5, CCL-2 and -5, and CXCL1 was decreased. p65 and IκBα protein nuclear translocation and degradation were impaired	Jiang et al. (2016)
	Shikimic Acid	*Artemisia absinthium* L., Asteraceae Bercht. and J.Presl	*In vitro,* IL-1β induced SW1353 human chondrocytes	0, 0.1,1,5,10, and 20 mM	0.1 mM	MAPK pathway	You et al. (2021)
			*In vivo,* ACLT surgery rats	20 mM in 100 μL *via* intra-articular injection	20 mM		
	Sinomenium	*Sinomenium acutum* (Thunb.) Rehder a& E.H.Wilson, Menispermaceae	*In vitro,* IL-1β induced human chondrocytes	10 mM	10 mM	AMPK/mTOR signaling pathway	Chen et al. (2016)
			*In vivo,* ACLT surgery mice	5 mg/kg *via* intra-articular injection for 4 or 8 w	5 mg/kg		
	Tetrahydrohyperforin	*Hypericum perforatum* L., Hypericaceae Juss	*In vivo,* mice indeced by intra-articular injection of collagenase solution	6 g/kg *via* oral administration once weekly for 6 w	6 g/kg	The levels of LC3-II, Beclin-1 and Atg5 were increased and The level of *p*-mTOR was decreased	Zhang et al. (2018b)
			*In vitro*, rats chondrocytes	50, 100, 150 and 200 μmol/L	50 μmol/L		

## 2 Autophagy-Mediated Cartilage Homeostasis and OA

Chondrocytes are the most dominant cells in cartilage tissue and play an important role in maintaining matrix integrity, and abnormal chondrocyte function is closely related to the development of OA. Chondrocytes play an important role in maintaining cartilage metabolic homeostasis by maintaining the stability of cartilage tissue and ECM (Kim and Blanco, 2007; Caramés et al., 2010; Charlier et al., 2019; Rim et al., 2020). When certain pathological factors act on the cartilage matrix and alter its structure, chondrocytes respond accordingly. However, the ability of articular chondrocytes to maintain normal cartilage matrix structure and integrity is limited and decreases with age (Martel-Pelletier et al., 2016). Molecular changes associated with aging of articular cartilage, as well as mechanical and inflammatory factors, can lead to elevated levels of reactive oxygen species (ROS), induce mitochondrial damage and ERS, severely impair chondrocytes and their ability to regulate ECM release, and may ultimately trigger the cascade of chondrocytes apoptosis (Wu et al., 2014; Bolduc et al., 2019). Dysfunction occurring in chondrocytes, including decreased survival (Loeser et al., 2016), inadequate ECM production (Mobasheri et al., 2017) and excessive activation of proteases (Kim et al., 2014) also accelerate cartilage degradation and disrupt joint microarchitectural integrity, leading to the development of OA.

**FIGURE 1:**
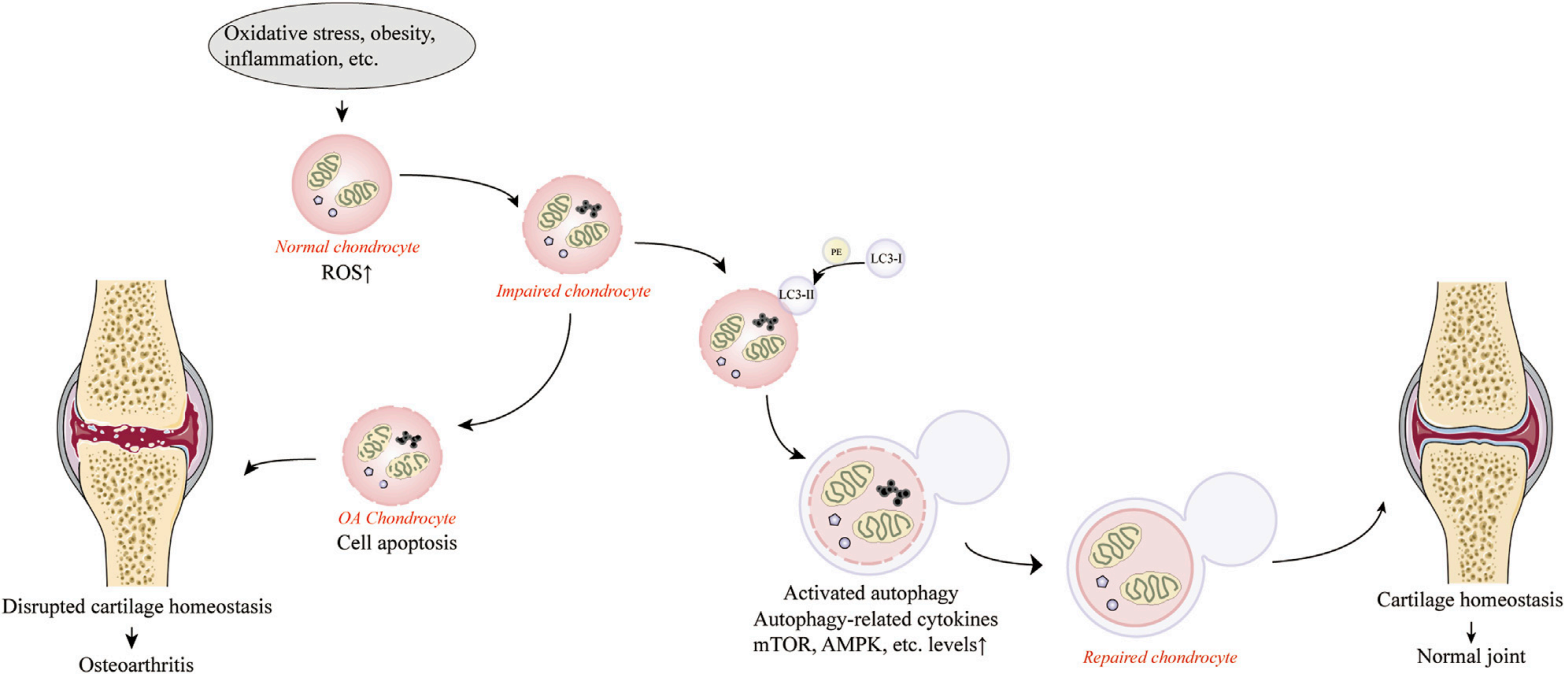
Activation of autophagy against osteoarthritis by maintaining cartilage homeostasis. Produced using Servier Medical Art (smart.servier.com).

Autophagy is an evolutionarily highly conserved degradation system that relies on the degradation of dysfunctional organelles and biomolecules by lysosomes under the regulation of autophagy-associated genes (ATG) to remove protein aggregates and dysfunctional organelles to maintain cellular homeostasis and protect cells from apoptosis (Levine and Kroemer, 2008; Mizushima et al., 2008; Nakamura and Yoshimori, 2017). When cells are exposed to abnormal physiological conditions, such as external stress, nutrient deficiency, hypoxia and ERS, autophagy is activated and plays a key role in regulating energy and nutrition and maintaining energy metabolism in the body (Duan et al., 2020). It was found that autophagy has a protective effect on cells in an inflammatory environment by regulating the body’s energy and nutrients and maintaining the body’s energy metabolism (Nakamura and Yoshimori, 2017; Yu et al., 2018). The most common marker of autophagy activation is the conversion of microtubule-associated protein 1A/1B-light chain 3 (LC3) from LC3-I to LC3-II, a lipidated form of the former protein that allows LC3-II to adhere to the phagocytic membrane during autophagosome formation and extension (Mizushima and Yoshimori, 2007). Under normal cellular conditions, most LC3 proteins are expressed as cytoplasmic LC3-I, which is converted to LC3-II upon autophagy activation by binding to diethanolamine phosphate (PE) and dispersing in the outer and inner membranes of autophagosomes, and this conversion is widely used as an indicator of autophagy activation (Jiang et al., 2016; Ansari et al., 2017). Autophagy deficiency or lack of autophagy activation induces apoptosis (Hara et al., 2006). It has been found that the expression of autophagy regulatory factors and autophagy-related proteins was observed to be downregulated in articular cartilage isolated from OA animals or humans, accompanied by increased chondrocyte apoptosis, suggesting that impaired autophagy is a contributing factor in the development of OA (Taniguchi et al., 2009; Caramés et al., 2010; Vinatier et al., 2018). In addition, the capacity of cells to degrade damaged components by activating autophagy is limited, and excessive oxidative stress can exceed the limits of autophagy, leading to a severe decrease in autophagic flux and impaired function, which can lead to cellular senescence and apoptosis, resulting in the progression of OA (Roca-Agujetas et al., 2019). In early stage of OA, autophagy in chondrocytes and cartilage tissue is confirmed by the upregulation of the autophagy-associated protein LC3. However, the transient activation of autophagy is shown to be only a compensatory response to cellular stress. In late stage of OA, the compensatory response fails to offset oxidative stress and causes structural cellular damage accompanied by inhibition of autophagy (Portal-Núñez et al., 2016; Tang et al., 2020). Nevertheless, the activation of autophagy during the early development of OA is still positive for chondrocyte survival. When autophagy is activated, damaged mitochondria can be removed and intracellular ROS are reduced, protecting chondrocytes from the negative effects of OA. There is evidence that enhanced autophagy in chondrocytes can slow the progression of OA by affecting intracellular metabolic activity (Caramés et al., 2010; Barranco, 2015; Caramés et al., 2015; Luo et al., 2019). Consequently, autophagy plays an irreplaceable role in protecting chondrocytes from oxidative stress (Ansari et al., 2018b). Figure 1 provides an overview of the mechanism of maintaining cartilage homeostasis through regulation of autophagy.

## 3 Phytochemicals for the Treatment of OA by Promoting Autophagy

**FIGURE 2:**
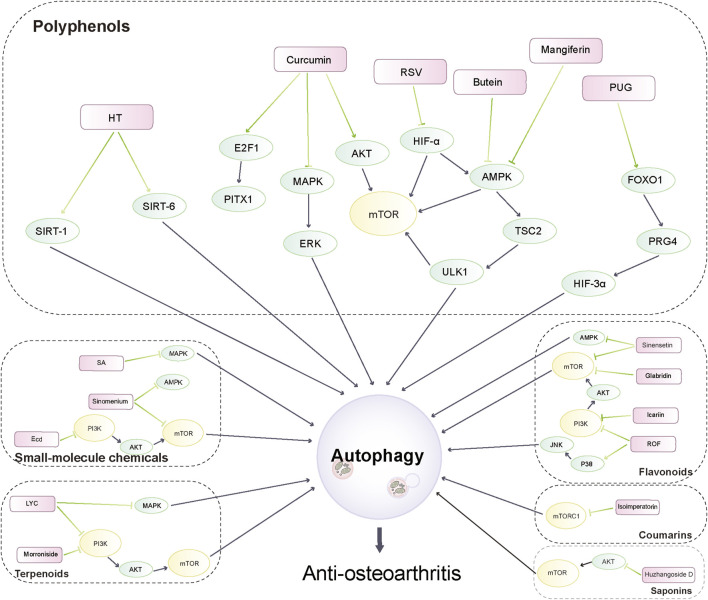
Autophagy pathways of phytochemicals in anti-osteoarthritis.

Phytochemicals from traditional medicinal plants are inexpensive and widely available, and can exert anti-inflammatory and antioxidant effects with good pharmacological activity. In recent years, various phytochemicals have been used in the prevention and treatment of various diseases by modulating autophagic targets, such as cardiovascular diseases and cancer (Hong et al., 2020; Li et al., 2020; Luo et al., 2020). Therefore, it is expected that phytochemicals derived from medicinal plants may retard the development of OA by mediating autophagy. There is increasing evidence that phytochemicals can also target autophagic pathways to function as complementary and alternative drugs for OA treatment, and their mechanisms related to the treatment of OA through the autophagic pathway are receiving increasing attention (Hu et al., 2020; Liu et al., 2020; Yu et al., 2021). In this review, we have selected phytochemicals that have the potential to activate autophagic pathways mediating cartilage homeostasis against OA in recent years. The anti-osteoarthritis activity of these phytochemicals and their mechanistic effects are described in the following sections. Figure 2 illustrates the autophagic pathways of phytochemicals against OA.

### 3.1 Polyphenols

Polyphenols, a large family of naturally occurring organic compounds characterized by multiples of phenol units, are widely distributed in numerous plants including vegetables, fruits, and botanical drugs (Salucci et al., 2017). It has been found that polyphenols are able to treat oxidative stress, inflammation and neurological diseases by mediating the autophagic pathway, and it has also been shown that polyphenols help to fight against OA (Varela-Eirín et al., 2020; Valsamidou et al., 2021). Here, we will summarize the existing scientific evidence gathered from *in vitro* and *in vivo* studies which support the beneficial effects of polyphenols on ameliorating OA by mediating the autophagic pathways.

#### 3.1.1 Curcumin

Curcumin, a polyphenol derived from *Curcuma longa* L*.*, has anti-inflammatory and antioxidant properties and functions as an anti-inflammatory therapeutic agent in Chinese medicine (Goel et al., 2008). Several studies have found that curcumin can treat OA by mediating autophagy-related pathways and thus improving the status of chondrocytes in an *in vitro* OA model. Li et al. treated mice primary chondrocytes with curcumin and observed that curcumin reduced the expression of autophagy markers LC3-II and Beclin-1 through suppressing mitogen-activated protein kinase/extracellular signal-regulated kinase 1/2 (MAPK/ERK1/2) signaling pathway, thereby inhibiting interleukin-1beta (IL-1β)-induced chondrocyte apoptosis and activation of cysteine aspartate protease-3 (Caspase-3), exerting its anti-inflammatory and anti-apoptotic effects (Li et al., 2017a). Similarly, Chen et al. also found that the curcumin pretreatment reduced IL-1β-induced apoptosis in rat primary chondrocytes through activation of autophagy and inhibition of nuclear factor-κ b (NF-κB) signaling pathway (Chen et al., 2021). In addition, curcumin has been found to activate autophagy and thus improve the function of chondrocytes *in vivo* models of OA. By establishing aging spontaneous OA and the destabilization of the medial meniscus (DMM) surgery-induced OA models in mice, Zhang et al. found that curcumin treatment promoted autophagy by suppressing the protein kinase B/mechanistic target of rapamycin kinase (AKT/mTOR) signaling pathway enhanced autophagy and reduced apoptosis and cartilage loss (Zhang et al., 2018a). Yao et al. established a rat model of OA by feeding a high-fat diet and injected curcumin into the knee joints of OA rats and found that the chondroprotective effect of curcumin may be achieved in part through inhibition of AKT/mTOR pathway autophagy (Yao et al., 2021). Besides, there is a study confirming that curcumin has been used clinically and has been shown to improve pain in patients with mild to moderate OA. But the limitations of curcumin being used in the clinic are mainly due to its low bioavailability and unstable metabolism in the body (Panahi et al., 2014). Curcumin showed low absorption and poor stability *in vivo* and *in vitro* environments (T1/2 < 5 min; F < 1%) (Yang et al., 2007). In addition, its solubility in water is limited, with the maximum solubility in pH 5.0 aqueous buffer only 11 ng/ml (Chen et al., 2015). It showed that orally administered curcumin at a dose of 1 g/kg only had 0.5 μg/ml maximum serum concentration, indicating only 1% oral bioavailability (Maiti et al., 2007). Current strategies to improve the bioavailability of curcumin are mainly to change the delivery method of curcumin, to modify it chemically or to combine it with other drugs (Prasad et al., 2014). In conclusion, curcumin, as an active ingredient derived from a natural drug that promotes autophagy, may have great promise in the treatment of OA through autophagy-mediated cartilage homeostasis.

#### 3.1.2 Hydroxytyrosol

Olive oil is the main source of fat in the Mediterranean diet (Granados-Principal et al., 2010). Studies on animal models of arthritis (Musumeci et al., 2013; Rosillo et al., 2014; Silva et al., 2015) and some preliminary trials (Bohlooli et al., 2012; Takeda et al., 2013) suggest that treatment with olive oil or olive phenol-rich extracts may be beneficial for patients with OA. Hydroxytyrosol (HT), the main phenolic in *Olea europaea* L, has been of interest for its potent antioxidant capacity, and studies have been conducted to identify the health benefits of HT in the prevention or treatment of cardiovascular disease, diabetes and cancer (Granados-Principal et al., 2010; Hu et al., 2014). Relevant *in vitro* experiments revealed that HT can regulate autophagy and thus improve chondrocyte function. Cetrullo et al. found experimentally that HT increases autophagy markers through the induction of the Sirtuin-1 (SIRT-1) pathway, thus protecting human primary chondrocytes from DNA damage and cell death due to oxidative stress increased by hydrogen peroxide (Cetrullo et al., 2016). It has been shown that HT increased autophagy in chondrocytes and preosteoblasts and protected them from cell death and oxidative damage (Aparicio-Soto et al., 2015). The results of Zhi et al. showed that HT may promote chondrocyte autophagy through Sirtuin-6 (SIRT6) and decreased Tumour Necrosis Factor alpha (TNF-α)-induced interleukin-1β (IL-1β) and interleukin-6 (IL-6) levels, thus protecting chondrocytes from TNF-α-induced inflammatory response and oxidative stress-induced DNA damage and cell death (Zhi et al., 2018). Such evidence suggests the potential of HT in preventing chondrocytes from being affected by OA through the relevant autophagic pathway. HT, a hydrophilic phenolic antioxidant, has broad potential for mediating autophagy in the treatment of OA (Cornwell and Ma, 2008).

#### 3.1.3 Resveratrol

Resveratrol (RSV), an active substance derived from *Vitis vinifera* L, has long been used in traditional Chinese medicine, and some studies have demonstrated that RSV inhibits the inflammatory response to OA in both *in vivo* and *in vitro* models (Zhang et al., 2020). Recently, *in vitro* and *in vivo* studies have also revealed that RSV can improve chondrocyte function by mediating autophagy-related pathways, thereby delaying OA progression. Qin et al. demonstrated experimentally that intra-articular injections of RSV upregulated the expression of hypoxia-inducible factor-1 alpha (HIF-1*α*) and hypoxia-inducible factor-2 alpha (HIF-2*α*) by up-regulating the AMP-activated protein kinase (AMPK)/mTOR signaling pathway, thereby promoting chondrocyte autophagy and delayed the degeneration of articular cartilage in a DMM-induced OA model (Qin et al., 2017). Additionally, RSV has been used clinically and has been shown to improve pain conditions and knee function in patients with OA (Nguyen et al., 2017; Wong et al., 2017; Hussain et al., 2018). It is notable that there are some limitations to the utilization of resveratrol. First, the solubility of resveratrol is relatively low, 0.05 mg/ml when dissolved in water, and in addition, resveratrol is chemically unstable when exposed to the intrinsic enzymatic and PH environment of the gastric and intestinal tract, which reduces the low bioavailability after oral administration (Francioso et al., 2014; Robinson et al., 2015). However, RSV still has the potential to improve chondrocyte homeostasis in OA progression by mediating autophagy, thus emerging as a new therapeutic approach for OA.

#### 3.1.4 Butein

Butein is a polyphenol derived primarily from *Butea monosperma* (Lam.) Kuntze. Plant extracts containing butein are traditionally used in Ayurvedic and Unani India as well as in Chinese medical systems to treat various human ailments. (Wang et al., 2014). Butein has been shown to inhibit the pro-inflammatory effects of IL-1β on chondrocytes (Zheng et al., 2017). Ansari treated IL-1β-induced human chondrocytes with butein and showed that butein activated autophagy through suppressing the AMPK/Tuberous sclerosis complex 2 (TSC2)/Unc-51-like kinase 1 (ULK1)/mTOR pathway and inhibited IL-6 expression in human chondrocytes, thereby protecting chondrocytes from OA (Ansari et al., 2018a). It has been found that Butein binds to human serum albumin (HSA) through hydrophobic interaction, thus successfully penetrating into the cell membrane to exert pharmacological effects (Padmavathi et al., 2017). As a potential therapeutic agent to improve chondrocyte homeostasis and thus treat and/or prevent OA through autophagy, butein deserves further investigation.

#### 3.1.5 Mangiferin

Mangiferin, a natural polyphenol derived mainly from *Mangifera indica* L*.*, is able to exert anti-inflammatory and antioxidant effects (Sellés et al., 2015), while it has been suggested that mangiferin may promote bone repair in endochondral osteogenesis by activating autophagy (Bai et al., 2018). Li et al. applied mangiferin treatment to reverse oxidative stress induced by tert-butyl hydroperoxide (TBHP) and alleviate chondrocyte apoptosis and ECM degradation in an *in vitro* experiment. *In vivo* experiments likewise confirmed that mangiferin could exert therapeutic effects on DMM-induced OA mice by activating autophagy. Their findings showed that mangiferin decreased mTOR activity and promoted phosphorylation of AMPK to activate autophagy in a time- and dose-dependent manner (Li et al., 2019b). This evidence demonstrates the ability of mangiferin to mitigate chondrocyte apoptosis and ECM degradation by regulating the autophagic pathway, thus exerting an anti-osteoarthritic effect. A clinical study found the concentration of mangiferin in plasma reached 38.64 ± 6.75 ng/ml approximately 1 h after oral administration of 0.9 g of mangiferin, with an apparent elimination half-life (t1/2) of 7.85 ± 1.72 h, further demonstrating its potential application in clinically mediated autophagy for the treatment of OA (Hou et al., 2012).

#### 3.1.6 Delphinidin

The natural compound anthocyanins extracted from *Aristotelia chilensis* (Molina) Stuntz are used to treat OA and several diseases (Kong et al., 2003). Among the various anthocyanins, delphinidin is a special class of polyphenols that protects chondrocytes and inhibits the progression of OA by mediating relevant cellular pathways against oxidative stress (Haseeb et al., 2013). Delphinidin has been reported to inhibit IL-1-induced matrix metalloproteinases (MMPs) expression in human articular chondrocytes via suppressing NF-κB and ERK/MAPK pathways (Wongwichai et al., 2019). Lee et al. applied delphinidin to hydrogen peroxide (H_2_O_2_)-treated human chondrocytes and found that delphinidin could activate autophagy by up-regulating nuclear factor erythroid 2-related factor 2 (NRF2) and NF-κB pathways to protect chondrocytes from oxidative stress generated by H2O2 (Lee et al., 2020). Therefore, delphinidin may protect chondrocytes by inducing autophagy and thus stop the progression of OA, providing a new idea for the treatment of OA. The high hydrophilicity and instability of delphinidin may restrict its use in clinical practice, so it requires strategies to be developed to increase its bioavailability (Legeay et al., 2019).

#### 3.1.7 Punicalagin

Punicalagin (PUG) is a hydrolyzable polyphenol extracted from *Punica granatum* L. with a wide range of biological activities and cytoprotective effects (Yan et al., 2016; Foroutanfar et al., 2020). PUG has been reported to play a protective role in inhibiting apoptosis, and related studies have also shown that pomegranate glucoside is beneficial in the treatment of OA (Yu et al., 2019; Foroutanfar et al., 2020). Both *in vivo* and *in vitro* studies have shown that PUG can exert anti-osteoarthritic effects through activation of autophagy. Liu et al. found that PUG promotes autophagy through the forkhead box O1/proteoglycan 4 (FOXO1/Prg4)/HIF3*α* axis, inhibits inflammation and extracellular matrix degradation, and attenuates inflammatory damage caused by lipopolysaccharide (LPS) in rat chondrocytes (Liu et al., 2021). In addition, Kong injected PUG in a mouse DMM model and showed that PUG increased antioxidant enzymes and reduced the degradation of apoptotic proteins and ECM by mediating autophagy, thus effectively stopping the progression (Kong et al., 2020). Although preclinical studies have shown that PUG can reduces chondrocyte apoptosis and ECM degradation through mediating autophagy, its solubility is low and insufficient for effective use after oral administration, which greatly limits its clinical application (Cerdá et al., 2005). However, the bioavailability of PUG can be promoted using micronization techniques therefore PUG has the potential to fight OA by mediating autophagy, but further experimental confirmation is needed (Nyamba et al., 2021).

#### 3.1.8 (-)-Epigallocatechin 3-Gallate

(-)-Epigallocatechin gallate (EGCG) is the most abundant bioactive polyphenol in *Camellia sinensis* (L.) Kuntze and has been shown to have anti-inflammatory and antioxidant effects (Luk et al., 2020). Related *in vitro* experiment found EGCG can attenuate extracellular matrix degradation and inflammation by inhibiting MMPs and TNF-α activation (Rasheed et al., 2009). In addition, Huang et al. observed that intra-articular injection of EGCG reduced cartilage degradation in an anterior cruciate ligament transection (ACLT)-induced OA rat model by regulating the expression levels of autophagy-related marker proteins, namely, decreasing mTOR expression and enhancing LC3, beclin-1, and p62 expression, thereby reducing chondrocyte inflammation and apoptosis (Huang et al., 2020). It has been shown that there is no toxicity in rats consuming up to 500 mg/kg/day doses of EGCG dietary (Isbrucker et al., 2006). And the bioavailability of EGCG at a dose of 800 mg/day showed a significant increase in healthy participants compared to a dose of 400 mg/day (Chow et al., 2003). Therefore, EGCG may be an alternative treatment for OA because of its high bioavailability and its ability to improve chondrocyte apoptosis and cartilage degeneration in OA rat models.

#### 3.1.9 Chlorogenic Acid

Chlorogenic acid (CGA) is a natural polyphenolic compound from *Bauhinia macrantha* Oliv. with antioxidant properties that have been found to protect against oxidative stress by activating multiple signaling pathways in chondrocytes (Hoelzl et al., 2010; Chen et al., 2011). Zada et al. found that CGA protects against H2O2-induced oxidative stress by mediating autophagy while regulating antioxidant pathways such as NRF2 and NF-κB pathways, thereby protecting human chondrocytes from apoptosis and slow down the progression of OA (Zada et al., 2021). A study reported that the apparent bioavailability of oral CGA in rats was 27–33% (Farah et al., 2008). These results suggest that CGA can be used for drug development in OA by modulating autophagy for targeted treatment of OA.

### 3.2 Flavonoids

In recent years, researches have confirmed the beneficial effects of flavonoids to health, including their use in conditions related to autophagy, such as neurodegenerative diseases (Dai et al., 2021a; Pérez-Arancibia et al., 2021). A large number of experiments have shown that flavonoids and its derivatives can improve chondrocytes and thus combat against OA, which makes it possible to apply flavonoids in clinical studies (Basu et al., 2018; Ansari et al., 2020).

#### 3.2.1 Icariin

Icariin, a flavonoid compound extracted from *Epimedium sagittatum* (Sieb and Zucc.) Maxim*.*, has been shown to slow down the progression of OA by inhibiting the NF-κB pathway to attenuate the inflammatory response of chondrocytes (Hua et al., 2018) and by inhibiting the expression of MMP-13 and pro-inflammatory cytokines in chondrocytes (Zeng et al., 2014; Pan et al., 2017a; Sun et al., 2018). Related *in vivo* and *in vitro* experiments revealed its ability to protect chondrocytes from OA by activating autophagy. The results of Mi et al. showed that icariin protects chondrocytes from OA by inhibiting TNF-α-induced NF-κB signaling pathway to activate autophagy and significantly reduce apoptotic markers. In addition, icariin exerted the same effect as the Pyrrolidinedithiocarbamic acid (PDTC, NF-κB inhibitor) control, but it exerted a better anti-inflammatory effect in inhibiting the activation of NF-κB signaling pathway (Mi et al., 2018). Tang et al. administered icariin intraperitoneally to DMM surgery induced OA rats and found that it attenuated OA in a dose-dependent manner by downregulating phosphatidylinositol 3-kinase (PI3K)/AKT/mTOR signaling to activate autophagy in chondrocytes (Tang et al., 2021). Icariin has a low bioavailability due to its poor absorption, which poses additional limitations to its clinical application (Li et al., 2015). However, it has been found that the bioavailability of icariin can be improved by pharmacological methods (Zhang et al., 2012). Therefore, the studies on the activation of autophagy to maintain the homeostasis of chondrocytes by icariin are of guidance for the prevention and treatment of OA.

#### 3.2.2 Baicalin

Baicalin, a principal flavonoid extracted from roots of *Scutellaria baicalensis* Georgi, has a wide range of biological activities such as anti-inflammatory and antioxidant (Wang et al., 2007; Lee et al., 2015), and some studies have shown that baicalin plays a role in improving OA progression (Pan et al., 2017b; Yang et al., 2018). Li et al. used baicalin to treat IL-1β-treated human OA chondrocytes, and the results showed that baicalin increased the promotion of autophagy flux and attenuated IL-1β-induced chondrocyte apoptosis and ECM degradation (Li et al., 2020). However, the bioavailability of baicalin was found to be very low (2.2%), therefore, how to improve the bioavailability of baicalin is still a problem (Xing et al., 2005). Nevertheless, it is undeniable that baicalin is promising in regulating the autophagic pathway against chondrocyte apoptosis and endochondral homeostasis against OA.

#### 3.2.3 Glabridin

Glabridin is a flavonoid derived from *Glycyrrhiza glabra* L. that exerts powerful antioxidant effects by removing free radicals and has been found to be comparable to vitamin E in terms of its antioxidant capacity (Fuhrman et al., 2000; Simmler et al., 2013). It has been used to prevent and treat pathological changes associated with free radical oxidation, such as cellular senescence (Veratti et al., 2011). In addition, glabridin has been found to enhance the function of osteoblasts thereby preventing osteoporosis and various bone and joint diseases (Choi, 2011). Dai et al. found a significant increase in LC3-II levels in human chondrocytes after the administration of glabridin, suggesting that it could protect them from oxidative stress, apoptosis by enhancing autophagy. *In vivo* experiments revealed that glabridin could activate autophagy, which is to reduce mTOR expression, and increase LC3-II levels attenuated ACLT-induced apoptosis in OA rats, thereby delaying their cartilage degeneration (Dai et al., 2021b). The clinical application of glabridin is limited due to its poor water solubility and therefore low bioavailability, but studies have shown that this situation can be improved by combining it with *β*-Lactoglobulin. (Wei et al., 2018). These results indicate the potential of glabridin in OA prevention and treatment as a potential drug for OA.

#### 3.2.4 Rhoifolin

Rhoifolin (ROF) is a flavanone derived from *Rhus succedanea* L. that exhibits significant antioxidant and anti-inflammatory effects in a variety of diseases (Fang et al., 2020; Peng et al., 2020). *In vitro*, ROF attenuated osteoclast-stimulated osteolysis by inhibiting MAPK and NF-κB signaling pathways (Liao et al., 2019). Yan et al. showed that ROF significantly blocked phosphorylation of P38/JNK and PI3K/AKT/mTOR pathways to enhance autophagy and protected rat chondrocytes from IL-1β-induced inflammation and apoptosis. *In vivo*, intra-articular injection of ROF also significantly improved cartilage damage in a rat OA model, affirming the potential role of ROF in OA treatment by regulating autophagy (Yan et al., 2021). However, the optimal dose of ROF for *in vivo* application is unclear and needs to be further investigated.

#### 3.2.5 Eupatilin

Eupatilin is a flavonoid derived from *Artemisia absinthium* L. that exerts anti-apoptotic and anti-inflammatory effects in a variety of diseases (Choi et al., 2008; Cai et al., 2012). Recent studies suggest that eupatilin may be useful in arthritis (Jeong et al., 2015; Kim et al., 2015). Lou et al. found that eupatilin attenuated IL -1β-induced apoptosis in rat chondrocytes by modulating autophagy-related protein levels, that is, by increasing senstrin2 and decreasing mTOR, which activated autophagy in a dose-dependent manner (Lou et al., 2019). Eupatilin at 33–85 mM exerts moderate toxic effects on human and mice cancer cells, but more eupatilin toxicity studies on chondrocytes are needed (Zater et al., 2016).

#### 3.2.6 Sinensetin

Sinensetin is a polymethoxylated flavonoid derived from *Citrus* L. with potent anti-inflammatory activity (Han Jie et al., 2020). Sinensetin has been shown to mediate the AMPK/mTOR signaling pathway to promote autophagy and thereby treat liver cancer (Kim et al., 2020). *In vitro* experiments by Zhou et al. showed that Sinensetin activated the AMPK/mTOR signaling pathway in a time- and dose-dependent mannersignificantly improved the autophagy function of mice chondrocytes and inhibited TBHP-induced apoptosis in mouse chondrocytes. *In vivo* experiments yielded similar results, and Sinensetin protected against DMM-induced mice (Zhou et al., 2021b). These results provide evidence that Sinensetin could be a potential candidate for the treatment of OA.

### 3.3 Terpenoids

Terpenoids are the largest class of natural products, most of which are of plant origin, and their biological properties, mainly anti-proliferative and apoptotic, have been shown to play an important role in cancer prevention and health promotion through the promotion of autophagy (Martel et al., 2019; El-Baba et al., 2021). Therefore, plant-derived terpenoids components will provide a novel approach for mediating autophagy in the treatment of OA.

#### 3.3.1 Morroniside

*Corni Fructus* (CF) is one of the most important botanical products in traditional Chinese medicine and its compound has been used clinically for a long time in the treatment of OA (Chen et al., 2014a). In latest years, some compounds extracted from CF such as cyclic enol ether glycosides and active ingredients have been found to have antioxidant and anti-inflammatory effects. Morroniside, an water-soluble iridoid glycoside derived from CF, has a variety of bioactive substances with antioxidant and anti-inflammatory effects (Park et al., 2009; Wang et al., 2010; Chen et al., 2014b). It has been shown in many *in vivo* and *in vitro* experiments that morroniside improves chondrocyte function by modulating autophagy to slow OA progression. Xiao et al. conducted *in vitro* experiments with human chondrocytes and showed that morroniside decreased the level of LC3 conversion in chondrocytes, thus promoting chondrocyte proliferation, survival and matrix synthesis. Interestingly, morroniside also inhibited the autophagic response of chondrocytes by promoting PI3K/AKT/mTOR signaling, while increasing the activity of AKT and mTOR. However, overexpression of autophagy genes in the experiment enhanced the positive regulation of cell proliferation, survival and matrix synthesis by morroniside, suggesting that activation of autophagy facilitates the protective effect of morroniside on chondrocytes (Xiao et al., 2020). Furthermore, Yu et al. established a mouse model of OA by DMM surgery and used primary mouse chondrocytes induced by morroniside treatment with IL-1β as *in vitro* subjects. Morroniside was found to slow down the progression of OA by inhibiting the NF-κB signaling pathway and suppressing the expression of MMP-13, Caspase-1 and NOD-like receptor family pyrin domain containing 3 (NLRP3) (Yu et al., 2021). Similarly, Cheng et al. treated human osteoarthritic chondrocytes with morroniside for *in vitro* experiments and found that morroniside could promote cell survival and matrix synthesis, and also induced an experimental OA model in rats by anterior cruciate ligament severance combined with medial meniscectomy (Hayami et al., 2003; Zhao et al., 2014), and found that intra-articular injection of morroniside reduced cartilage damage in an experimental model of OA rats (Cheng et al., 2015). Therefore, some drugs with autophagy-activating effects may serve as effective supplements to morroniside in the treatment of OA.

#### 3.3.2 Lycopene

Lycopene (LYC), a lipid-soluble carotenoid naturally found in *Solanum lycopersicum* L, is a potent antioxidant with potential chondroprotective effects (Sachdeva and Chopra, 2015; Wang et al., 2016). It is shown *in vitro* experiments by Wu et al. that LYC may enhance autophagy and inhibit H_2_O_2_-induced inflammation and apoptosis in rat chondrocytes by suppressing mTOR levels in part through inhibition of MAPK and PI3K/AKT/NF-κB axis (Wu et al., 2021), thus LYC has potential therapeutic implications for mediating autophagy in the treatment of OA.

#### 3.3.3 Celastrol

Celastrol is a natural triterpenoid found in traditional Chinese medicine botanical drugs *Tripterygium wilfordii* (Yang et al., 2006). Owing to its powerful anti-inflammatoryand antioxidant properties, celastrol has been used to treat a variety of diseases (Kannaiyan et al., 2011). Feng et al. found that celastrol restored IL-1β-induced inhibition of autophagy in rat chondrocytes by inhibiting the NF-κB signaling pathway, increased the expression levels of LC3-II and Beclin-1, and ameliorated IL-1β-induced apoptosis in rat chondrocytes. In addition, celastrol decreased IL-1β-stimulated phosphorylation of IκBα and P65. The therapeutic effect of celastrol on OA articular cartilage was also confirmed in the ACLT rat OA model (Feng et al., 2020). Celastrol is poorly water soluble, but it has been studied to effectively improve its bioavailability through galactosylated liposomes (Chen et al., 2020a). These results suggest that celastrol has potential in the prevention and treatment of OA.

### 3.4 Coumarins

Coumarins are different species of plant-derived secondary metabolites that have anti-inflammatory and anti-apoptotic properties and have recently been found to treat cancer by mediating autophagy (Guo et al., 2019; Rodríguez-Hernández et al., 2020). Accordingly, it is possible that these plant-derived coumarins mediate the autophagic pathway for the treatment of OA.

#### 3.4.1 Isoimperatorin

Isoimperatorin is an linear furanocoumarin isolated from the *Angelica dahurica* (Wei and Ito, 2006). It has been demonstrated that isoimperatorin may exert anti-osteoarthritic effects by inhibiting COX-2 production in a mouse model (Moon et al., 2008). Ouyang et al. found that isoimperatorin activated autophagy by downregulating the mTORC1 signaling pathway in a dose-dependent manner in mouse chondrocytes, and downregulated the expression of MMP13, Runt-related transcription factor 2 (Runx2), and Vascular endothelial growth factor (VEGF) expression of inflammatory factors, thereby ameliorating articular cartilage degeneration in a mouse DMM model (Ouyang et al., 2017). Thus, isoimperatorin activates autophagy in a dose-dependent manner to down-regulate the level of inflammatory factors in cartilage and thus anti-osteoarthritis. Isoimperatorin was found to have toxic effects on human tumor cell lines, but more data is needed on its effects on chondrocytes (Kim et al., 2007).

#### 3.4.2 Isopsoralen

Isopsoralen is a linear furanocoumarin isolated from the fruit of *Cullen corylifolium* (L.) Medik. and has various pharmacological effects such as anti-inflammatory, anti-oxidative stress (Li et al., 2019c; Zhang et al., 2019). Isopsoralen has been found to promote chondrocyte differentiation (Li et al., 2012). A study by Chen et al. showed that isopsoralen significantly enhanced autophagic flux by reducing the expression of LC3-II and sqstm1/p62, which resulted in a protective effect against il-1β-induced apoptosis in rat chondrocytes (Chen et al., 2020b). isopsoralen deserves consideration as a therapeutic agent for OA.

### 3.5 Saponin

Saponins originating from different plants have also been found to exert anti-inflammatory and antioxidant activity through the mediation of autophagy and thus be used in the treatment of various diseases (Zhou et al., 2019; Luo et al., 2020). Saponins have potential in mediating autophagic pathways in the treatment of OA.

#### 3.5.1 Astragaloside IV

Astragaloside IV (AST) is a unique active saponin compound extracted from *Astragalus mongholicus* Bunge, which has been found to protect against IL-1β-induced joint inflammation and cartilage damage (Wang and Chen, 2014). Liu et al. treated chondrocytes from OA patients induced by IL-1β with AST and demonstrated that AST increased chondrocyte autophagic flux, reversed the expression of LC-3II/I and p62, significantly inhibited IL-1β-induced apoptosis, and maintained cell viability (Liu et al., 2017). AST may be a new candidate for the treatment of OA because of its potential to mediate autophagy to regulate cartilage function. However, the gastrointestinal absorption of AST is very low, with a bioavailability of 2.2% in rats, thus limiting its clinical application (Gu et al., 2004). More research is needed to make it better applied in the clinical treatment of OA.

#### 3.5.2 Huzhangoside D

Huzhangoside D is a saponin isolated from *Clematis graveolens* Lindl, the latter being used to treat inflammation (Kawata et al., 1998). Zhang et al. treated an ACLT-induced KOA rat model with huzhangoside D and found that huzhangoside D may activate autophagy through downregulation of AKT and mTOR signaling pathway activity to activate autophagy, thereby upregulating the levels of autophagy-associated proteins beclin-1, ATG5, ATG7 and LC3. Meanwhile the levels of pro-inflammatory cytokines including TNF-α, IL-6 and IL-1β were decreased and the levels of anti-inflammatory cytokines such as IL-10 were increased, and the damage to cartilage structures and the function of cartilage in KOA rat models were restored to some extent (Zhang et al., 2021).

### 3.6 Small-Molecule Chemicals

In addition to the above phytochemicals, a number of small-molecule chemicals derived from plants, such as various natural compounds and compound analogues, have also been found to enhance autophagy to treat various diseases (Jang et al., 2019; Wu et al., 2020). These plant-derived small molecules would be able to mediate autophagy to treat OA, but more research is needed.

#### 3.6.1 *β*-ecdysterone

*β*-ecdysterone (Ecd) is an estrogen analog isolated from *Achyranthes bidentata* with various physiological effects such as anti-fatigue, pro-proliferative and hypolipidemic (Xu et al., 2018). It was found that Ecd inhibited IL-1β-induced apoptosis and inflammation in rat chondrocytes by inhibiting the NF-κB signaling pathway (Zhang et al., 2014). Tang used Ecd to intervene in a rat OA model established by monoiodoacetic acid (MIA), and the results showed that Ecd may induce chondrogenesis through the down-regulation of PI3K/AKT/mTOR signaling pathway induced chondrocyte autophagy and downregulated the expression of PI3K, mTOR, and caspase-3, thereby reducing OA-like symptoms (Tang et al., 2020). Evidence related to Ecd improving chondrocyte homeostasis through mediated autophagy requires further examination.

#### 3.6.2 Dihydroartemisinin

Dihydroartemisinin (DHA), a semisynthetic derivative of Artemisinin (ART), has fewer side effects than ART, the main phytochemical extracted from *Artemisia annua* L. (Tu, 1999). It has been shown that DHA can inhibit estrogen deficiency-induced osteoporosis and osteoblast remodeling (Zhou et al., 2016). Jiang et al. have found *in vitro* experiments that DHA inhibits TNF-α-induced NF- κB pathway activation and promote autophagy in rat chondrocytes thereby inhibiting chondrocyte catabolism and inflammatory factor levels, thus DHA has potential in improving OA through enhanced autophagy (Jiang et al., 2016). Currently, it has been found that the compounding of hydroxypropyl-beta-cyclodextrin (HPβCD) with DHA increases the solubility and stability of DHA, which makes it possible to apply DHA to clinical trials (Ansari et al., 2009).

#### 3.6.3 Shikimic Acid

Shikimic acid (SA) is a hydroaromatic compound extracted from *Artemisia absinthium L*, which has been shown to have significant anti-inflammatory effects (Kolodziej et al., 2008). Another study showed that SA can inhibit osteoclastogenesis by inhibiting NF-κB and MAPK pathways, suggesting its potential role in the treatment of OA (Chen et al., 2018). *In vitro* and *in vivo* experiments have recently demonstrated that SA can delay OA progression by enhancing autophagy-mediated cartilage homeostasis. You et al. found that SA inhibited IL-1β-induced mitogen activation by inhibiting phosphorylation of ERK, p38, JNK, p65, and MAPK and NF-κB pathway activation and attenuated the expression of inflammatory and apoptotic factors, such as iNOS, COX2, MMPs and ADAMTS5, thereby alleviating IL-1β-induced inflammatory responses, dysregulation of cartilage anabolism and catabolism, and autophagic damage in human chondrocytes. In addition, *in vivo* experiments revealed that SA reduced cartilage erosion and proteoglycan loss in ACLT rats, thus providing new evidence for water-soluble SA to treat and prevent the development of OA (Steimer et al., 2015; You et al., 2021).

#### 3.6.4 Sinomenium

Sinomenium (SIN) is a natural alkaloid extracted from the medicinal plant *Sinomenium acutum* (Thunb.) Rehder and E.H.Wilson*,* which decreases the protein level of MMP-13, a marker of cartilage degradation in rats, thereby resisting cartilage degradation, and blocks collagen-induced arthritis through NF-κB signaling and downregulates the expression of MMP13 (Ju et al., 2010; Sun et al., 2014). Chen et al. found that SIN improved IL-1β-induced degradation of the extracellular matrix in mouse chondrocytes at least in part through activation of autophagy. Similarly, by intra-articular injection of CM-SIN and GelMA in ACLT surgery-induced mice, SIN was also found to improve cartilage matrix degradation, at least in part, by inducing autophagy *in vivo* (Chen et al., 2016).

#### 3.6.5 Tetrahydrohyperforin

Tetrahydrohyperforin (IDN5706) is a tetrahydro derivative of hyperforin, one of the main active components of *Hypericum perforatum* L. extracts, which has many properties, including anti-inflammatory and anti-tumor properties (Koeberle et al., 2011). *In vitro* studies by Zhang et al. demonstrated that IDN5706 decreased the levels of MMP-13 and IL-6 in OA rat chondrocytes. At the same time, the levels of LC3-II, Beclin-1 and Atg5 were increased and the levels of *p*-mTOR were decreased. IDN5706 was also found to attenuate the degeneration of OA rat model induced by intra-articular injection of collagenase solution articular cartilage through activation of autophagy (Zhang et al., 2018bbib_Zhang_et_al_2018a).

## 4 Summary and Prospect

In the development of OA, the activation of autophagy has positive significance for the survival of chondrocytes, and autophagy as a therapeutic target for OA has a broad clinical application prospect. Targeted application of drugs to regulate chondrocyte autophagy levels is expected to provide more options for the clinical treatment of OA. In recent years, plant extracts and phytochemicals isolated from the former have gradually gained popularity and attracted widespread attention. They have definite anti-osteoarthritic ability, which is mainly achieved by mediating relevant autophagy-related pathways, and improving chondrocyte autophagy. Given the cost-effectiveness and wide availability of phytochemicals, and the growing awareness of their role in promoting chondrocyte autophagy and thus treating OA, research on improving OA is rapidly increasing. Phytochemicals emerge as an important source for the development of novel OA therapeutics, providing a valuable source of OA therapeutics, lead compounds or adjuvants for the discovery of new drugs that exert multifaceted biological activities in maintaining human health and preventing disease. However, except from curcumin and RSV (Chin, 2016; Nguyen et al., 2017), most of the current experimental designs are confined to preclinical studies such as cellular and animal experiments, and it remains unclear whether they can be applied in the clinics. Furthermore, there are still many issues to be solved in this field. First, *in vitro* experiments have demonstrated curcumin at concentrations of 25 μM and above is cytotoxic to primary chondrocytes after 5 days in culture, but most phytochemicals still lack validated clinical trials to demonstrate their toxicity (Clutterbuck et al., 2013). Second, some studies have demonstrated a dose-effect effect of curcumin, EGCG and HT in the improvement of chondrocyte function, so it is possible that different doses of phytochemicals have different efficacy in the treatment of OA, and there are still no comparative studies on the effectiveness of other phytochemicals with different doses (Chowdhury et al., 2008; Oliviero et al., 2013; Zhi et al., 2018). Third, it has been argued that phytochemicals such as curcumin, resveratrol, and EGCG may be pan-assay interference compounds (PAINS), that is, by interfering with various reactions in the assays, such as chemical aggregation, the presence of a reactive michael acceptor, and fluorescence activity, thus making the assays readout appear false positive rather than through specific compound/target interactions. Moreover, its experimental results are strongly influenced by the experimental conditions, such as the purity of the phytochemical extracts (Baell, 2016). The pharmacological effects of these phytochemicals in a variety of diseases have attracted widespread attention from researchers as a panaceas, but this invalid metabolic panaceas (IMPS) may lead to a waste of research resources in drug development, so further exploration in the control of experimental conditions and development is needed to minimize the effect of false positives (Bisson et al., 2016). Autophagy can be used as a protective mechanism to degrade and remove damaged organelles and proteins, and thus can be used to treat various aging-related diseases (Ren and Zhang, 2018). In oxidative stress environments, autophagy is activated to eliminating intracellular ROS to protect cells from apoptosis, as well as to prevent tissue inflammation by downregulating inflammation-related signals, reducing inflammatory cytokine expression, and promoting apoptotic corpse clearance (Levine et al., 2011; Yun et al., 2020). Current studies have shown that activation of autophagy can protect chondrocytes from oxidative stress and inflammation by down-regulating the levels of inflammation and oxidative stress-related proteins such as iNOS and MMPs and upregulating autophagy-related factors such as mTOR and AMPK to maintain the structural integrity of the cartilage matrix against early OA (Duan et al., 2020), but the role of autophagy in different periods of OA is different, and further studies are needed on the mechanism of autophagy activation on advanced OA and its effects. Besides, it has been shown by another study that curcumin has potential therapeutic potential for diseases such as viral infections by modulating APE1 and thus affecting the common redox response in the body, and therefore is not a PAINS (Li et al., 2019a). Since Apurinic/apyrimidinic endonuclease 1 (APE1) can promote autophagy, it is possible that phytochemicals like curcumin could enhance the regulation of APE1 by targeting autophagy to treat OA, thus avoiding becoming an IMPS and the failure of drug development due to false positives in its pharmacological effects, but the role of phytochemicals in mediating autophagy in different periods of OA and the specific molecular mechanisms of their anti-osteoarthritis function need to be further investigated, and further experimental demonstration on the existing basis is needed (Li et al., 2019d; Tang et al., 2019). Obviously, our review has the limitation that there is a risk of obtaining false-positive results. However, in the context of developing natural products with the potential to target the treatment of OA, our review remains a summary and critical evaluation based on the available evidence and provides a basis for further research and development. Therefore, the combined efforts of more relevant researchers and experts are needed to conduct systematic experimental design and experimental analysis and to establish an effective evaluation system so that phytochemicals can more effectively benefit patients with OA. It is hoped that increasingly extensive and well-designed pharmacological and clinical studies will enable safer and more rational use of these ancient botanical drugs based on conclusive evidence.
